# Optimization of the emergency obstetric and neonatal care network in Benin through expert-based sub-national prioritizations

**DOI:** 10.3389/fgwh.2024.1265729

**Published:** 2024-06-03

**Authors:** Zeynabou Sy, Yaniss Guigoz, Michel Brun, Thierry Tossou Boco, Venance Vodungbo, Thierry Lawalé, Theodore Soude, Yawo Agbigbi, Nicolas Ray

**Affiliations:** ^1^ GeoHealth Group, Institute of Global Health, Faculty of Medicine, University of Geneva, Geneva, Switzerland; ^2^ Institute for Environmental Sciences, University of Geneva, Geneva, Switzerland; ^3^ Technical Division, United Nations Population Fund (UNFPA), New York, NY, United States; ^4^ Programme de la Santé Familiale, OMS-Benin, Cotonou, Benin; ^5^ Direction de la Programmation et de la Prospective, Ministère de la Santé, Cotonou, Benin; ^6^ Agence Nationale des Soins de Santé Primaires, Cotonou, Benin; ^7^ United Nations Population Fund (UNFPA), Cotonou, Benin; ^8^ United Nations Population Fund (UNFPA), Lomé, Togo

**Keywords:** geographical accessibility, maternal health, AccessMod, EMOC, universal health coverage, Africa

## Abstract

**Introduction:**

To reduce maternal mortality by 2030, Benin needs to implement strategies for improving access to high quality emergency obstetric and neonatal care (EmONC). This study applies an expert-based approach using sub-national travel specificities to identify and prioritize a network of EmONC maternities that maximizes both population coverage and functionality.

**Methods:**

We conducted a series of workshops involving international, national, and department experts in maternal health to prioritize a set of EmONC facilities that meet international standards. Geographical accessibility modeling was used together with EmONC availability to inform the process. For women in need of EmONC, experts provided insights into travel characteristics (i.e., modes and speeds of travel) specific to each department, enabling more realistic travel times estimates modelled with the AccessMod software.

**Results:**

The prioritization approach resulted in the selection of 109 EmONC maternities from an initial group of 125 designated maternities. The national coverage of the population living within an hour's drive of the nearest EmONC maternity increased slightly from 92.6% to 94.1% after prioritization. This increase in coverage was achieved by selecting maternities with sufficient obstetrical activities to be upgraded to EmONC maternities in the Plateau and Atlantique departments.

**Conclusion:**

The prioritization approach enabled Benin to achieve the minimum EmONC availability, while ensuring very good geographical accessibility to the prioritized network. Limited human and financial resources can now be targetted towards a smaller number of EmONC facilities to make them fully functioning in the medium-term. By implementing this strategy, Benin aims to reduce maternal mortality rates and deliver effective, high-quality obstetric and neonatal care, especially during emergencies.

## 1 Introduction

Target 3.1 of the Sustainable Development Goals (SDGs) aims to reduce maternal mortality by 2030, with a global target for a maternal mortality ratio (MMR) below 70 per 100,000 live births (1). Achieving this target requires significant efforts to improve maternal health, particularly in countries with high MMR. In 2017, Benin had an MMR of 397 deaths per 100,000 live births (UI 291–570) (2), 32.7 neonatal deaths per 1,000 live births (UI 23–46.9) (3), and 20.74 (UI 18.52–23.23) stillbirths per 1,000 total births (4). The MMR of Benin increased to an estimated 523 deaths per 100,000 live births (IU 397–768) in 2020 (5).

One of the most important strategies to reduce this high maternal mortality ratio is to facilitate rapid access to maternities providing better quality emergency obstetric and neonatal care (EmONC) (6). Numerous studies have confirmed that reducing maternal and neonatal mortality must be accompanied by a reduction in travel times to the nearest maternity (7–9). However, timely geographic access to EmONC remains a real challenge for large segments of the population in many low-income countries (10).

To address the challenge of inadequate access to emergency obstetric care, the United Nations Population Fund (UNFPA) introduced an innovative approach in 2015 (10). This approach is designed to aid countries grappling with high maternal mortality ratios by prioritizing EmONC maternities capable of providing functioning and quality EmONC. The level of EmONC functionality is defined based on EmONC signal functions. There are a total of 7 signal functions for maternities that provide basic EmONC (BEmONC): administer parenteral antibiotics, administer uterotonic drugs, administer parenteral anticonvulsants, perform manual removal of the placenta, remove retained products, perform assisted vaginal delivery, and perform basic neonatal resuscitation (with bag and mask). Maternities providing complete EmONC (CEmONC) perform, in addition, blood transfusion and caesarean section. An EmONC maternity is considered functioning if services are available 24/7 and all of its signal functions were performed at least once in the three months before an EmONC assessment (11).

The approach is based on the observation that many low- and middle-income countries have designated (i.e., specified) a number of EmONC maternities that often largely surpasses the World Health Organization's (WHO) international norm for EmONC availability (6) which is at least 5 EmONC maternity facilities, including one CEmONC maternity, per 500,000 population. This situation rarely offers quality care because resources are spread too thinly over a large number of maternities, most being not functioning. Prioritizing a network of maternities capable of delivering quality EmONC can optimize the network's adequacy and timely geographic access through standardized indicators (12). These indicators recommend calculating travel time to the nearest EmONC maternity within the national network to determine the population living within 2-h travel time to EmONC. Our use of the term “network” follows UNFPA terminology (11), mainly reflecting referrals between BEmONC and CEmONC maternities, and does not imply that all EmONC maternities operate in a coordinated and interconnected manner.

For women who need to attend a maternity, travel means and associated average speeds may vary regionally, depending on transportation constraints and regional characteristics of infrastructure (e.g., roads) or the natural environment (presence of obstacles such as forests, wetlands, etc.) (13). In our earlier similar study in Togo (14), we used a national travel scenario assuming same travel characteristics in all rural areas. With the increasing availability of high-resolution geospatial data, it is now possible to account for these subnational specificities, with the help of national and local experts who can inform on subnational specificities related to travel characteristics and barriers to mobility.

Geographical accessibility is believed to be a major obstacle to access maternal and newborn health services in Benin (15), which, combined with other factors such as partner's education, economic status, marital status, and parity (16), represents a challenge towards decreasing the national MMR in the country (17). The Ministry of Health of Benin (MoH, thereafter) committed in 2017 to reduce maternal and newborn deaths by implementing UNFPA's EmONC maternities prioritization approach (18).

In addition to assessing the geographical accessibility to the national EmONC network in Benin, the purpose of this study was to demonstrate how the EmONC prioritization approach can be instrumental in prioritizing a high number of EmONC maternities that maximizes both population coverage and the potential for future functionality of the network. This study also reports, for the first time, the use of subnational travel specificities for women in needs of EmONC when conducting travel time accessibility modeling.

## 2 Methods

### 2.1 Study area

Located in West Africa, Benin has an estimated population of 11.554 million in 2018, about half living in rural areas (19). Based on the 2017–2018 Demographic and Health Surveys Report (20), the total fertility rate is estimated at 5.7 children per woman. The national percentage of births which occur in health facilities is relatively high at 84%, but varies in the 12 departments, from 61% (Borgou) to 99.2% (Littoral). Traditional birth attendants still play an important role in the country (21) and home births remain common in some departments such as Borgou (38%), Atacora (27%), Alibori (27%), Donga (20%) and Couffo (13%).

### 2.2 Approach for prioritizing EmONC maternities

**Figure 1:**
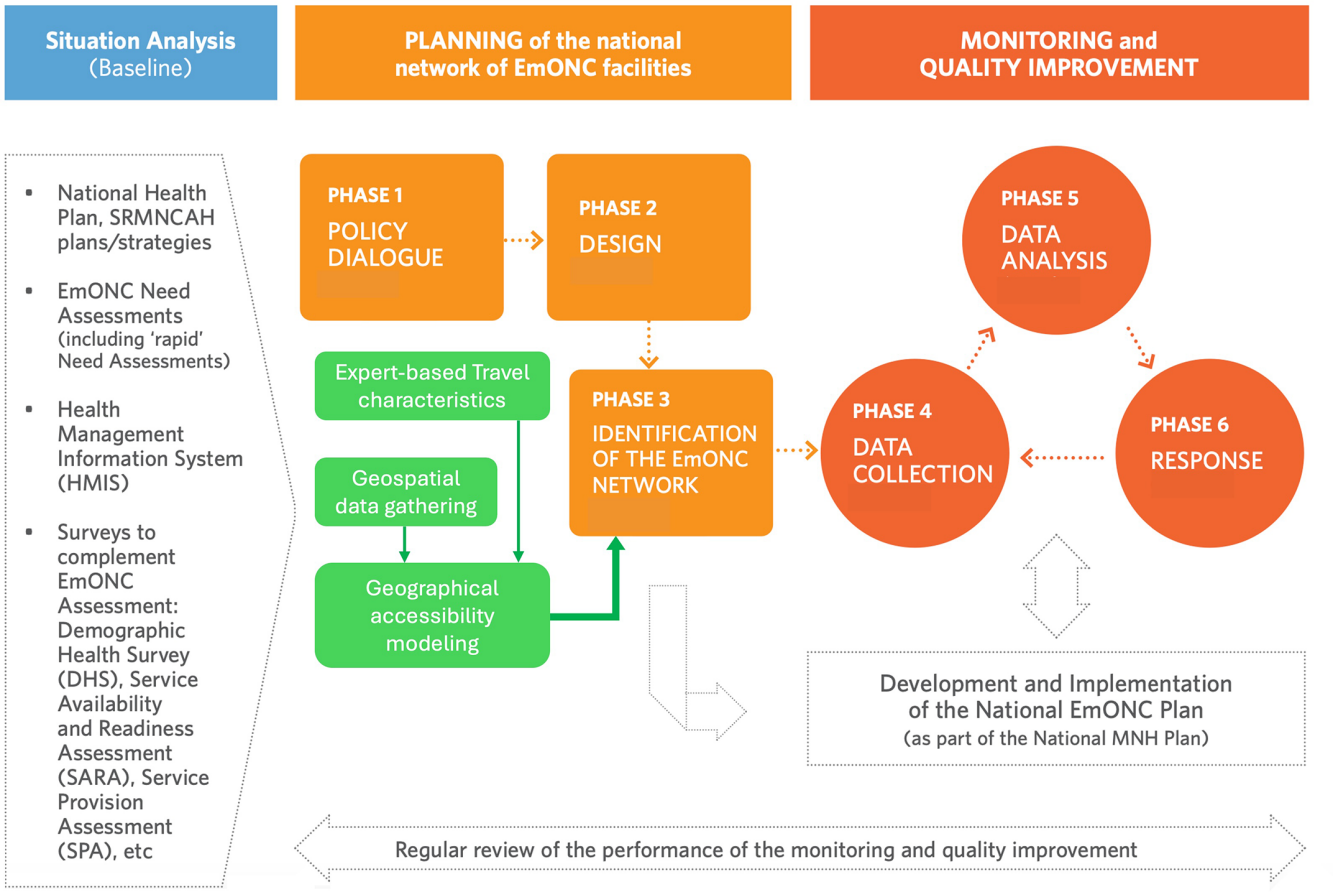
Overall process of the EmONC development approach, with the three phases leading to EmONC prioritization (in orange) and geographical accessibility modeling support (in green). Adapted from (11).

We followed the EmONC development approach recently described in a dedicated UNFPA implementation manual (22), and whose process and main six phases are illustrated in Figure 1. We briefly describe below how the first three phases were implemented in Benin and articulated with the geographical accessibility modeling. Phases 4–6 address iterative monitoring and quality improvement of the EmONC network, which is currently underway in Benin and not discussed in our study. During Phase 1 (Policy dialog), advocacy efforts for the development of a national network of EmONC maternities were undertaken to ensure the involvement and commitment of key political stakeholders and decision-makers in maternal and neonatal health (MNH) to the proposed principles and processes.

In Phase 2 (Design), MoH and other stakeholders, as well as international experts, gathered in an initial national workshop held in November 2017 to introduce the prioritization approach, discuss the needed data, confirm the prioritization approach, and obtain stakeholder buy-in to begin the process. Three decisions were taken: (1) to maintain a number of EmONC maternities that does not exceed the WHO minimum recommendation for EmONC availability, which is equal to 115 EmONC maternities in Benin in 2018; (2) to prioritize a set of EmONC maternities that maximizes the population that can reach the nearest prioritized EMONC maternity within one hour travel time by motorized transport (population coverage, thereafter), a more ambitious standard than the WHO recommendation of a maximum travel time of 2 h (6)—owing to the relatively small size of the country and the good condition of the road network; and (3) to keep all CEmONC maternities in the final prioritized group of EmONC maternities.

For Phase 3 (Identification of the EmONC network), a series of three regional 4-day prioritization workshops was held in November–December 2018 targeting three sets of departments: (i) Atlantic, Littoral, Ouémé, and Plateau, (ii) Collines, Couffo, Mono, and Zou, and (iii); Donga, Borgou, Alibori, and Atacora. Workshop attendance was about sixty participants who were usually departmental (i.e., at the level of the department) health directors, heads of the department's main health structures, and representatives of professional associations (midwives, nurses, etc.) and local communities. During these workshops, participants used the maps and results of the departmental accessibility models to define consensually a set of prioritized EmONC maternities for their department. In addition to geographical accessibility of maternities and population coverage, other factors considered in the prioritization process include obstetrical activity, EmONC functionality, and other types of potential barriers to health care access (e.g., financial). Empirical referral times between each BEmONC facility and its known reference CEmONC facility are also considered by the expert groups to inform their choices. Following the workshops, the prioritized network at departmental level were merged to allow analysis of national geographical accessibility.

### 2.3 Data preparation

#### 2.3.1 EmONC maternities

The rapid UNFPA EmONC assessment done in 2016 in Benin (23) revealed that 387 maternities routinely provided obstetric care, i.e., with more than 20 births/month (averaged over the previous 3 months at the time of the prioritization workshops). This monthly number of births rate is a minimum standard chosen by the MoH to define a health facility with regular obstetric activities in Benin. 125 of these maternities were designated by the MoH to provide EmONC (EmONC maternities, thereafter). In addition to the number of births and complications per month, the EmONC assessment also provided the level of EmONC functionality and the information on the provision of each EmONC signal function.

#### 2.3.2 Geospatial data

Spatial population distribution at 30-m resolution is from “Center for International Earth Science Information Network/Data for good at Meta” (CIESIN/Meta) (24) for 2018, which is an appropriate data set for use in accessibility modeling as it constrains the population within building footprints, unlike other datasets that disperse the population more broadly (25). To represent the 2018 population distribution, we adjusted the CIESIN/Meta population counts to match the 2018 departmental demographic projections from the *Institut National de la Statistique et de l'Analyse Economique* (20). Population adjustment was performed in AccessMod 5.6.42 (13, 26), where all populations residing on pixels classified as “barriers” are redistributed outside of these barriers.

Administrative boundaries (at national and departmental level), rivers, wetlands, and road network were provided by the *Institut Géographique National du Bénin* (IGN-BENIN). Roads are classified into 4 categories: primary roads, secondary roads, tracks and trails. Specific travel speeds were assigned to each road category. In some cases, and based on expert knowledge, different travel speeds were assigned to the same road categories due to departmental specificities. Major rivers were considered complete barriers to travel except where bridges were present (informed by the road network). Wetlands and water bodies may or may not be considered navigable depending on the department.

The land cover raster is from IGN-BENIN and contains 14 categories (see Supplementary Material S1). This land cover is used to determine off-road travel speeds, whether on foot or by motorized vehicles. Slopes were derived from the 90 m digital elevation model (DEM) from the Shuttle Radar Topography Mission of the US National Aeronautics and Space Administration (27).

**Figure 2:**
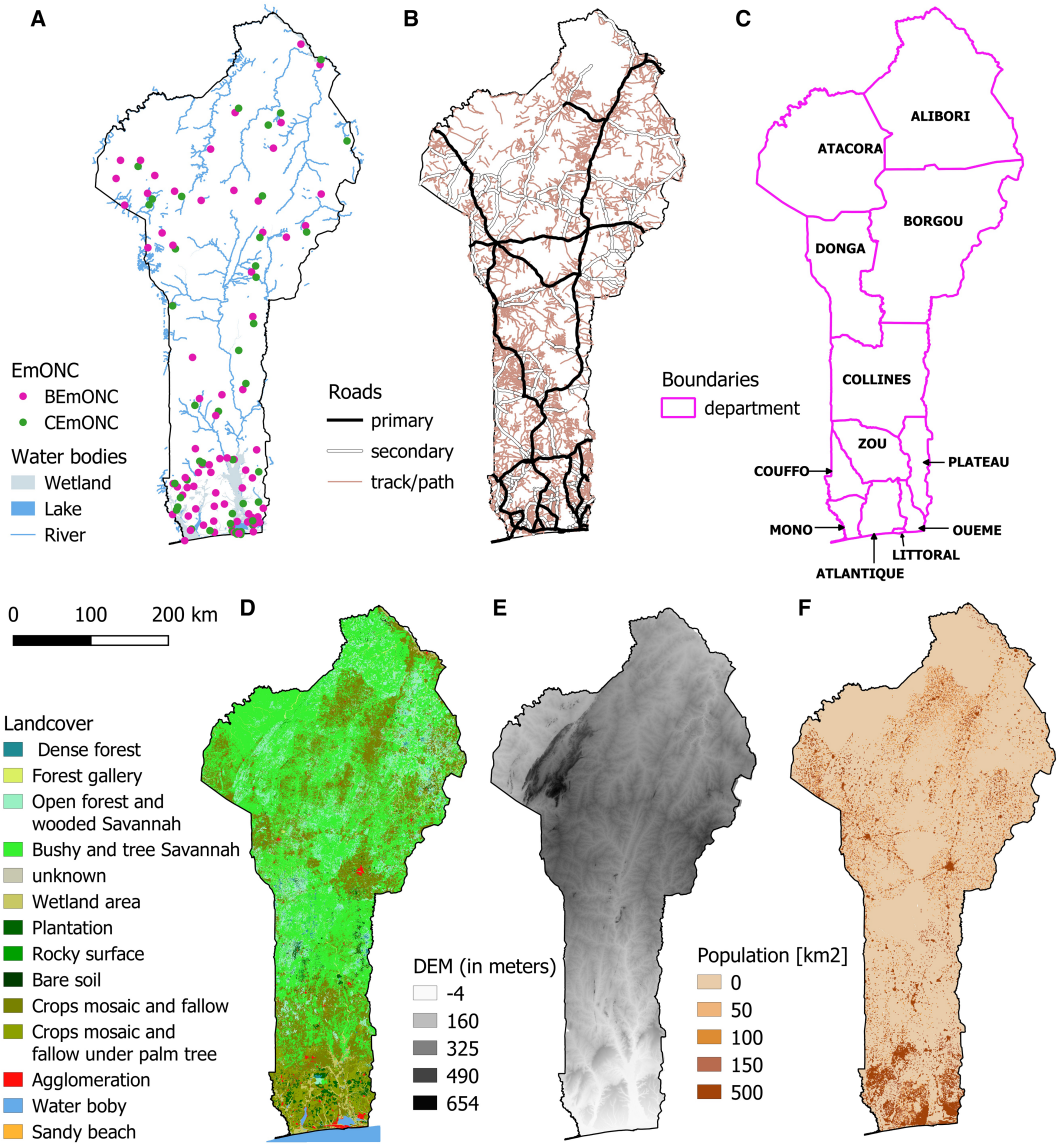
Geospatial layers used in the modeling of geographic accessibility in Benin: (**A**) EmONC facilities and water bodies, (**B**) road network, (**C**) department boundaries, (**D**) landcover, (**E**) digital elevation model, (**F**) population density.

Supplementary Table S1 gives a summary of all data sets with links for data download. We used QGIS 2.18 software (28) to prepare all geospatial data sets (Figure 2) that were projected in the WGS 84/UTM zone 31 N projection. The projected population, landcover and DEM were all resampled at 92 m resolution. Road, land cover, and river data were merged using AccessMod to create the “merged landcover” raster that is the basis for calculating travel times in AccessMod.

### 2.4 Modeling geographical accessibility to EmONC maternities

To assess the adequacy of each department with the WHO recommendation for EmONC availability, we calculated the minimum recommended number of EmONC maternities for each department. To estimate the travel time of the population to the nearest EmONC maternity, we used the *Accessibility* module of AccessMod. This module is based on a least-cost path algorithm that uses a travel scenario with the merged land use raster and produces a raster map of travel times to the nearest maternity. Walking speeds (towards the maternities) are corrected by the slope according to Tobler equation (29), which is not the case for the speeds of motorized vehicles. The means of travel and associated on- and off-road speeds were determined by consensus between national experts and departmental experts (Supplementary Material S1) following a standardized methodology (30). This process was carried out independently for each department to take into account departmental specificities. The same travel scenario was assigned to the departments with the same geographical characteristics, whose travel behavior can be considered very similar.

The assumptions underlying the accessibility modeling in Benin were that (i) use of motorcycles is frequent (31) and motorized off-road travel (mostly by motorcycles, but also by car) is possible, (ii) the nearest EmONC maternity (in terms of travel time) is used by the population, (iii) women in need of EmONC are preferentially going to a CEmONC facility if it is closer than the nearest BEmONC facility, otherwise they go to the nearest BEmONC facility and (iv) the travel scenario represents the situation in the dry season, which can last up to eight months in Benin.

To obtain the population coverage of the considered EmONC networks at both national and departmental levels, we used the *Zonal Statistics* module of AccessMod. Population coverage at the departmental level takes into account the influence of the catchment areas of the maternities in neighboring departments. We used the *Geographic Coverage* module of AccessMod to model the extent of catchment areas at a maximum travel time of 1 h for each maternity. Finally, we also calculated the mean travel time at national level and at departmental level, separately for the EmONC network before and after prioritization. This calculation was done for each department by: (1) multiplying the population raster by the travel time raster to obtain weighted (by population) travel times; (2) summing this weighted travel times contained in each cell by department, and dividing this number by the department population. Following the same procedure at national level, we calculated the mean national travel time.

## 3 Results

The prioritization process resulted in 109 prioritized EmONC maternities, which is 16 structures (13%) fewer than the initial set of 125 designated EmONC maternities. All of the 109 prioritized EmONC maternities had more than 20 deliveries/month, but some of them were not present in the initial set of 125 EmONC maternities.

### 3.1 Population coverage

Before prioritization, the 125 designated EmONC covered 92.6% of the total population located within 1 h travel from the nearest EmONC maternity. This percentage is 94.1% for the network after prioritization. The 387 maternities that perform more than 20 deliveries per month cover 97% of the population within 1 h travel time.

The two departments (Donga, Zou) where the number of prioritized EmONC maternities is below the minimum WHO-defined EmONC availability have very good population coverage of over 92%. Table 1 shows these figures in detail for each department. While in most departments the final number of prioritized EmONC maternities is less than or equal to the initial number of designated EmONC maternities, in 2 departments (Plateau, Atlantique) it is higher.

**Table 1:** Number of structures and coverage of the population within 1 h of travel of the various networks considered.

	Number of EmONC maternities expected according to standard	Maternities carrying out more than 20 deliveries per month		EmONC maternities designated before prioritization by the government		Functioning EMONC according to the 2018 rapid assessement		EmONC maternities prioritized by the government		Functioning EmONC maternities of the network after prioritization	
		Number	Population coverage	Number^a^	Population coverage	Number^a^	Population coverage	Number^a^	Population coverage	Number^a^	Population coverage
Plateau	7	23	99.9%	5 (2)	98.8%	1 (1)	94.6%	7 (3)	99.1%	2 (2)	97.5%
Ouémé	12	17	99.4%	15 (5)	98.6%	2 (2)	97.6%	12 (4)	97.7%	1 (1)	97.6%
Littoral	7	28	100%	12 (8)	100%	4 (4)	99.9%	8 (7)	100%	5 (5)	100%
Atlantique	16	50	98.7%	11 (3)	98.1%	2 (2)	95.6%	16 (4)	98.2%	3 (3)	97.9%
Mono	5	15	98.7%	8 (3)	98.9%	3 (3)	97.6%	5 (2)	98.1%	1 (1)	94.7%
Couffo	8	21	99.7%	9 (3)	97.4%	1 (1)	95.3%	8 (3)	99.7%	3 (3)	96.5%
Zou	9	50	94.2%	12 (3)	92.2%	0 (0)	62.3%	7 (3)	93.0%	2 (2)	88.8%
Collines	8	31	99.0%	9 (5)	89.7%	2 (2)	56.2%	9 (5)	97.0%	0 (0)	17.6%
Donga	6	34	98.8%	6 (2)	94.2%	1 (1)	28.6%	5 (2)	95.0%	2 (2)	89.9%
Borgou	14	44	92.6%	14 (7)	77.8%	4 (4)	54.9%	14 (6)	83.2%	5 (5)	57.0%
Alibori	10	38	91.0%	11 (4)	77.1%	3 (3)	59.6%	10 (4)	84.3%	3 (2)	48.3%
Atacora	8	36	98.4%	13 (5)	91.7%	2 (2)	46.7%	8 (4)	86.8%	3 (2)	58.4%
National	110	387	97.0%	125 (50)	92.6%	25 (25)	75.6%	109 (47)	94.1%	30 (28)	78.8%

^a^ The number in brackets indicates CEmONC only.

### 3.2 Functionality and geographical accessibility

**Figure 3:**
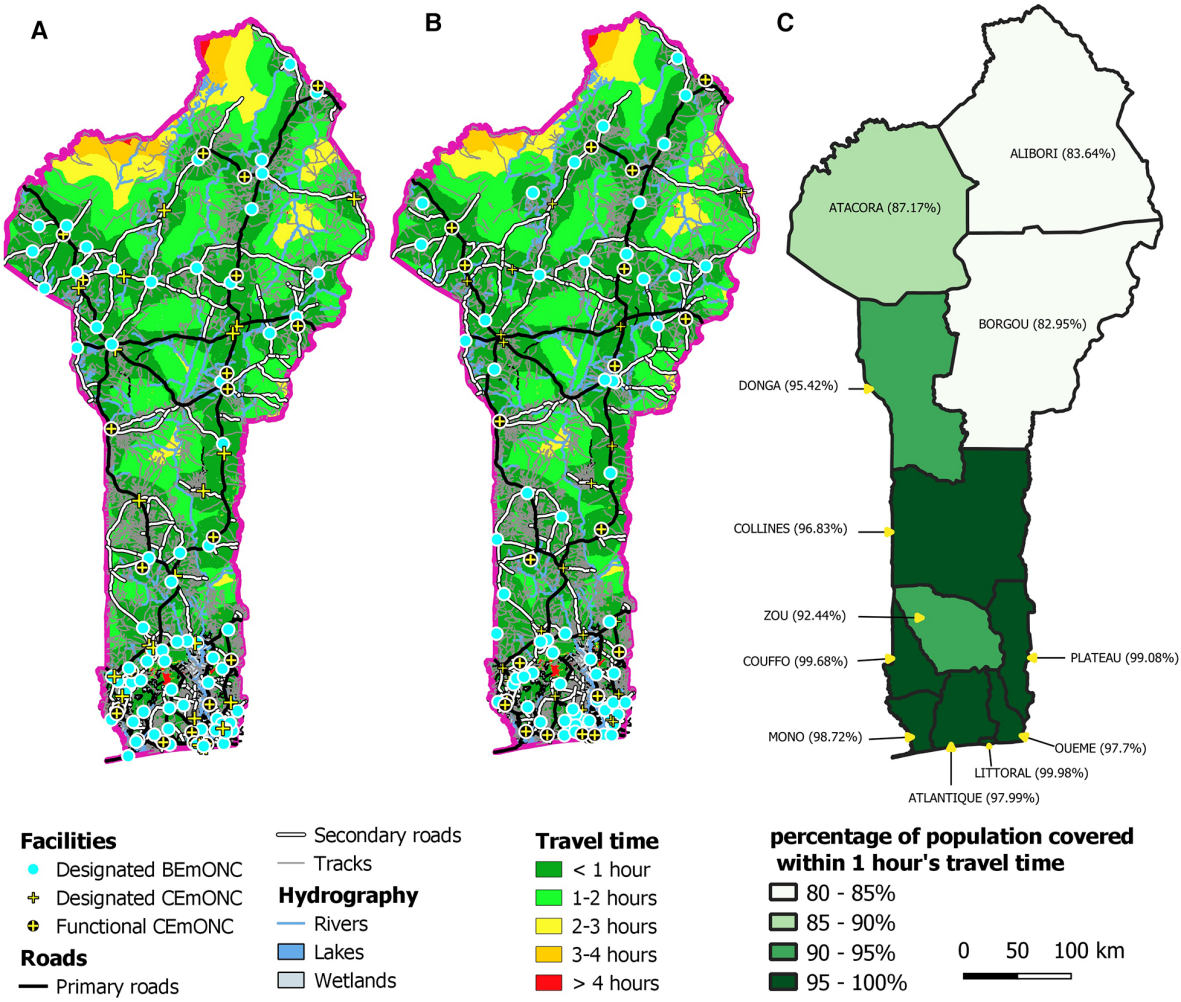
Travel time maps to designated EmONC maternities (**A**) before prioritization, (**B**) after prioritization. (**C**) Population coverage of the prioritized EmONC network at 1 h maximum travel time, for each department.

Analysis of data from the rapid EmONC assessment showed that only 25 EmONC maternities were functioning in 2018, which is 22% of all the designated EmONC maternities. Due to their preferred location in urban areas, these 25 functioning EmONC maternities cover 75% of the population. Maps of geographical accessibility (travel time) before and after prioritization and percentage coverage by department are shown in Figure 3.

### 3.3 Case studies: Atacora and Alibori departments

**Figure 4:**
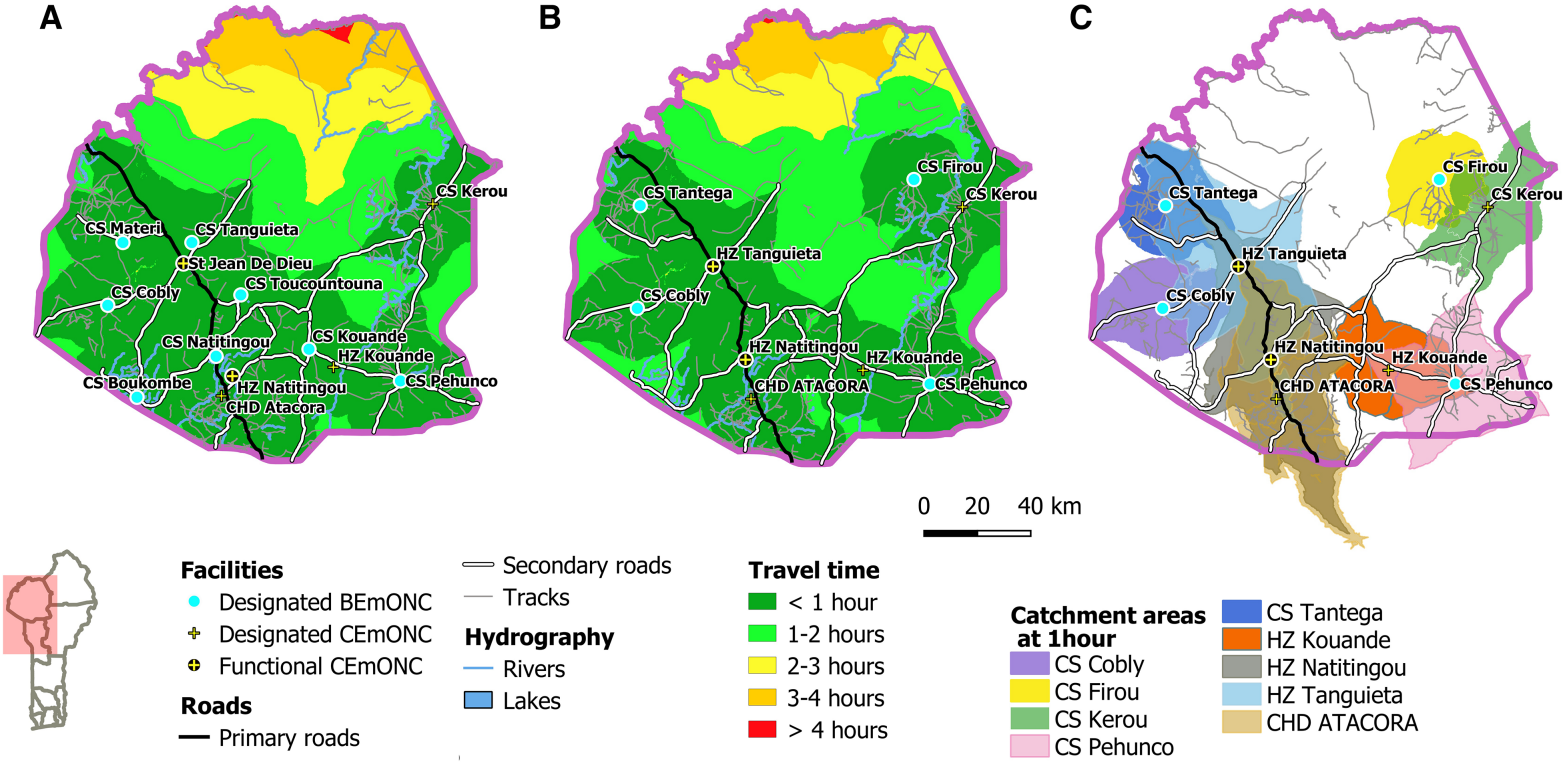
Accessibility maps to EmONC facilities in the Atacora region (**A**) before prioritization, (**B**) after prioritization. (**C**) One-hour catchment areas of each prioritized EmONC facility.

To illustrate how prioritization changes EmONC population coverage at the departmental level, we first show the accessibility of Atacora's EmONC maternities in Figure 4. In this department, the number of EmONC maternities decreases from 13 to 8 after prioritization. The population coverage decreases from 91.7% to 86.8% after prioritization, with pockets of low geographical accessibility in the north of the department in the area of the Pendjari national park. The EmONC catchments after prioritization (Figure 4C) are quite complementary.

**Figure 5:**
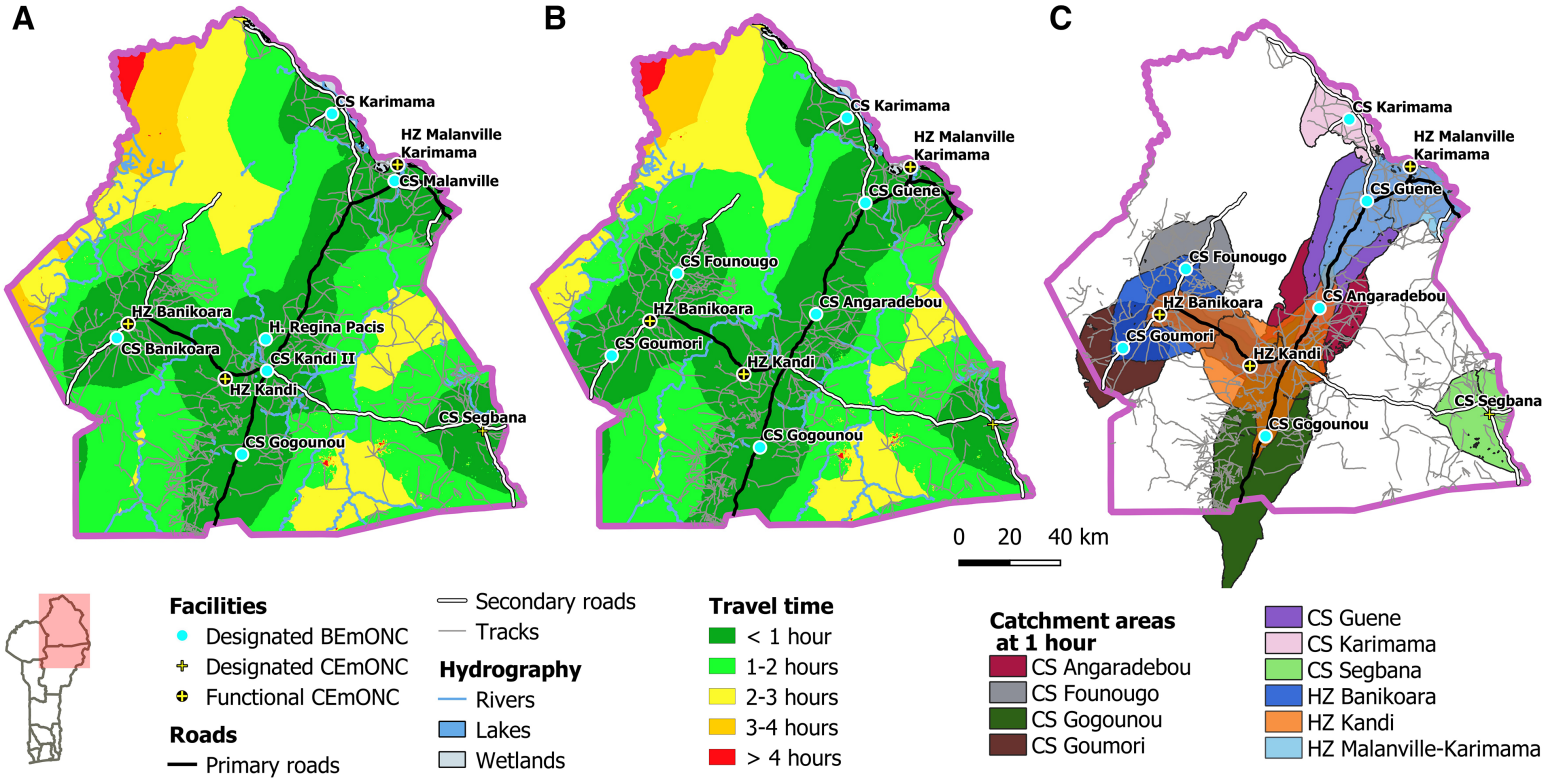
Accessibility map to EmONC facilities in the Alibori region (**A**) before prioritization, (**B**) after prioritization. (**C**) One-hour catchment areas of each prioritized EmONC facility.

The second example (Figure 5) is the department of Alibori, with a network of 11 EmONC maternities before prioritization and 10 after. Lower accessibility if found in the different forest areas (south, southwest, and east) and in the national park (north) of the department. Population coverage within 1 h travel time increases from 77.1% before prioritization to 84.3% after prioritization. This increase in population coverage despite a lower number of prioritized EmONC maternities is explained by the decision of the expert group to retain two maternities (CS Founougo and CS Goumori) that were not part of the initial designated EmONC network. Their inclusion in the final network allow to significantly improve the coverage of the population in the western part of the department. A similar scenario emerged for the departments of Couffo and Donga.

The catchment areas of the prioritized EmONC maternities (Figure 5C) partially overlap. These overlaps highlight that the 2 functioning CEmONC hospitals in the Alibori department (HZ Banikoara and HZ Kandi) are relatively close to several BEmONC maternities, indicating a travel time of less than 1 h when referrals are made from BEmONC to CEmONC maternities.

### 3.4 Overall impact at national and departmental levels

As shown in Table 2, the mean travel time has marginally decreased following prioritization at the national level, while the population coverage has increased. At the departmental level, the situation is more diverse, but it is essential to consider the influence of neighboring departments on the population covered, as a portion of this population may seek services in adjacent departments.

**Table 2:** Mean travel time comparison before and after prioritization, by department and nationally at 1 h travel time.

Administrative unit	EmONC network before prioritization			EmONC network after prioritization		
	Number of EmONCs	Mean travel time (minutes)	Population coverage (%)	Number of EmONCs	Mean travel time (minutes)	Population coverage (%)
Alibori	11	37	77.1	10	34	84.3
Atacora	13	29	91.7	8	34	86.8
Atlantique	11	13	98.1	16	12	98.2
Borgou	14	36	77.8	14	34	83.2
Collines	9	24	89.7	9	23	97
Couffo	9	14	97.4	8	12	99.7
Donga	6	27	94.2	5	26	95
Littoral	12	2	100	8	3	100
Mono	8	11	98.9	5	13	98.1
Oueme	15	8	98.6	12	9	97.7
Plateau	5	20	98.8	7	15	99.1
Zou	12	34	92.2	7	36	93
National	125	20.9	92.6	109	20.6	94.1

## 4 Discussion

The prioritization of the EmONC network in Benin was instrumental to optimize this network not only for slightly improving timely geographical accessibility to EmONC, but most of all for decreasing the number of EmONC maternities in that network. The approach resulted in the selection of 109 EmONC maternities, mostly from the 125 structures initially designated by the MoH. The theoretical coverage of the national population (at 1-h travel time) increased from 92.6% for the 125 designated EmONC maternities before prioritization to 94.1% for the set of 109 prioritized EmONC maternities. This prioritization achieved the goal of not exceeding the national WHO standard for the number of EmONC maternities (115 for Benin in 2018), with the majority of departments having prioritized a number of EmONC maternities equal to the WHO standard at departmental level. As a result, the MoH can focus its human and financial resources on a smaller number of EmONC maternities while maintaining a very high population coverage within 1-h travel time that is only 3 percentage points lower than the 97% coverage of all 387 maternities with more than 20 deliveries per month. It can also be noted that the small reduction of the mean national travel time after prioritization is an additional indicator of the positive impact of the EmONC network prioritization.

Modeling geographic accessibility was critical in determining which maternities to prioritize, as it provided experts with a quantitative assessment of population coverage and an opportunity to compare alternative scenarios. The regional workshops, which brought together experts from the different departments, led to a detailed and informed reflection on the specificities of each department in terms of travel characteristics and barriers to mobility. This resulted in various travel scenarios that reflect department-specific constraints on the modes of travel and associated average travel speeds for our target population. We note that these specificities would not have been captured in a national scenario that applies to everyone (15). This highlights the importance of working at a subnational level when defining travel scenarios. This is in sharp contrast to travel scenarios defined with broad assumptions applied to all sub-Saharan residents (32, 33). In addition, modeling and mapping individual EmONC catchments allowed experts to visualize overlapping catchments of neighboring EmONC maternities, which better informed prioritization decisions and provided additional insight into theoretical referral linkages between maternities.

In the Plateau and Atlantique departments, the number of prioritized maternities surpassed the initially designated EmONC facilities. This situation arose when the expert groups chose to extend the network by including supplementary maternity units, considering both their performance of more than 20 deliveries/month and their strategic geographical positions. Although these additional maternities were not officially designated as EmONC, their incorporation was deemed essential to enhance the network's coverage and accessibility. These maternities will receive the necessary equipment and personnel to operate at full capacity and deliver BEmONC services effectively within the next programmatic cycle.

It is important to mention that the population coverage statistics are theoretical and can only be considered meaningful if the network of EmONC maternities is fully functioning so that women with obstetric complications can receive rapid, high quality care in an efficient network. Coverage of the population by the 25 functioning EmONC maternities was determined to be 75% for 2018. Therefore, the primary operational goal is to increase the number of functioning EmONC maternities and increase the functioning network coverage from 75% to 94.1% when all prioritized EmONC maternities are functioning.

To achieve functionality of the prioritized EmONC network, the next step is to establish regular monitoring (every 3–4 months) of the entire prioritized network to monitor EmONC signal functions, identify resource gaps, and enable appropriate responses (22). This monitoring phase has started to be implemented in mid-2021 in Benin. Other countries that have already prioritized their EmONC network have been able to start this monitoring, including neighboring Togo (14). Initial feedback from Togo showed significant improvements in signal functions that are often deficient in many countries, such as the use of magnesium sulfate (eclampsia) or the suction cup. This feedback also highlighted the need for more funding to make the prioritized EmONC network fully operational. We can make the same observation in other countries such as Burundi and Madagascar, where the UNFPA's Maternal Health Thematic Fund has supported similar EmONC prioritization processes.

Regarding midwifery workforce and training, Benin employed 1,460 midwives in 2019, 877 of whom work in the public sector. There are two midwifery training centers, a national midwifery association (Association des Sages-Femmes du Bénin) and a national midwifery order (Ordre National des Sages Femmes du Bénin). The preliminary standard adopted during the national workshop in Benin states that a minimum of 4 midwives per EmONC maternity is needed to provide high quality care 24/7, and that each midwife must attend a maximum of 30 deliveries per month to ensure high quality of care. 49% of maternities in the prioritized network do not meet this standard in terms of minimum number of midwives. To close this gap, we calculated that 122 additional midwives would need to be deployed. With 26% of EmONC maternities in the network, particularly BEmONC maternities, having at least 3 signal function deficits, there are both quantitative and qualitative midwifery needs. Indeed, discussions with MoH officials during the situational analysis of the departmental workshops revealed a gap between the need for obstetric and neonatal skills and the skills acquired by midwives during their training. This situation, found in many countries, should alarm us in the design of the training curriculum for these key personnel and/or the way it is implemented.

Our study facilitated the establishment of a geospatial database and departmental travel scenarios for accessibility modeling, which can be highly valuable for optimizing other health services. By tailoring the travel scenarios to different target populations, a similar approach can be used to optimize, for example, vaccine coverage (34), the optimal location of HIV treatment centers or tuberculosis centers, or the deployment of community health workers to maximize their impact and reach (35). This can be facilitated in Benin by the national technical working group that has been set up following a dedicated AccessMod capacity building workshop carried out during the EmONC prioritization process.

Our data and analyzes have several limitations. We assumed that the geographic distribution of the population matched that of our target population of pregnant women with obstetric complications. However, heterogeneous distribution of fertility across the country could cause this spatial distribution to vary, which could have a slight upward or downward effect on the population coverage estimates. Road network data, while theoretically complete for the primary and secondary road networks, only partially capture the tertiary network of tracks and trails. This could lead to an underestimation of accessibility in some areas, particularly in rural departments (e.g., Atacora, Alibori, and Donga) known to have extensive tertiary networks. In urban areas, we did not specifically adjust travel speeds based on traffic volume or other urban constraints because we lack specific information. Accounting for these urban constraints could affect accessibility modeling in these areas, as shown in Bangladesh in particular (36). The “On Tackling In-transit delays for Mothers in Emergency” (OnTIME) consortium has already provided realistic travel times to EmONC maternities based on big data from Google product users within several large African cities (37, 38).

Travel scenarios developed based on expert knowledge allowed for a nuanced representation of departmental travel characteristics. However, these scenarios remain an approximation based on perceived modes and speeds of travel, and are subject to unknown uncertainties. Future studies in the country should ideally address these shortcomings by replicating expert knowledge elicitation or using complementary data such as vehicle tracking data at the national level. In addition, only the dry season was modeled, and the effects of the rainy season can alter the road network and off-road traffic, especially in rural departments where roads are of lower quality. Accounting for seasonality would help to better understand the differences between departments and identify the populations most at risk of significantly losing accessibility during the rainy season. Prioritizing an EmONC network could also give different results if a travel scenario for the rainy season is used.

Our study did not attempt to model by-passing behavior, in which the nearest EmONC maternity is avoided in favor of one farther away, as has been shown in several studies (37, 39, 40). Although this behavior was not quantified in Benin, it could contribute to an overestimation of actual geographic accessibility. In addition, borders with neighboring countries were assumed to be closed to cross-border travel to seek health services.

This study was mainly concerned with geographic access to health care. Other facets of access were only marginally discussed in the workshops. The issue of acceptability of care (respectful care) was raised in the discussions on quality of human resources. Financial barriers to accessing some EmONC maternities were highlighted by workshop participants during the discussion of referral linkages, suggesting that the problem exists.

A key strength of our study lies in the development of a prioritized EmONC network that ensures a high level of geographical accessibility, with over 94% of the population capable of reaching the nearest EmONC facility within a one-hour motorized travel time. The incorporation of field knowledge in the decision-making process at the department level is a novel aspect of our approach, enabling the development of six travel scenarios that reflect the unique sub-national characteristics of travel modes and average speeds. By integrating this information into the modeling of geographical accessibility and generating detailed travel time and catchment area maps, we empowered national and departmental decision-makers to make well-informed choices in prioritizing the EmONC network. The ongoing implementation of regular monitoring for EmONC facilities, along with the flexibility for future updates in geographical data and travel constraints, positions this approach as a mechanism to help transitioning the prioritized EmONC maternities into a fully functional EmONC network. While the reduction in the number of initially designated EmONC maternities was a pivotal step, the true challenge lies in enabling the prioritized network to deliver effective, high-quality obstetric and neonatal care, especially during emergencies. This includes establishing effective links between non-EmONC maternity units and the prioritized EmONC network.

## Data Availability

The raw data supporting the conclusions of this article will be made available by the authors, without undue reservation.

## In memoriam

Dr. Theodore Soude, co-author of this study, passed away tragically during the final stage of the manuscript preparation. Dr. Theodore Soude was a reference for maternal and neonatal health and EmONC in Benin and will be missed dearly.
