# Supplementary material for: Optimization of the emergency obstetric and neonatal care network in Benin through expert-based sub-national prioritizations
Source: Front Glob Womens Health. 2024 Jun 3;5:1265729. doi: 10.3389/fgwh.2024.1265729 (PMC11180813; doi:10.3389/fgwh.2024.1265729)
Supplement: Supplementary file 1 [file Datasheet1.docx]

**Supplementary material 1**

The following tables give the travel scenarios (speeds and modes of travel) for the 12 Departments of Benin. They are grouped by pairs of departments that features putatively similar travel modes and speeds. Speeds of 0 indicate complete barriers of movement.

1. Scenario for Alibori and Borgou departments

| **class** | **label** | **Speed (km/h)** | **mode** |
| --- | --- | --- | --- |
| 1 | Dense forest | 0 | WALKING |
| 2 | Forest gallery | 8 | MOTORIZED |
| 3 | Clear forest and wooded savannah | 20 | MOTORIZED |
| 4 | Tree and shrub savannah | 20 | MOTORIZED |
| 5 | Bare and sparse vegetation | 10 | MOTORIZED |
| 6 | Swamp | 5 | WALKING |
| 7 | Planting | 10 | MOTORIZED |
| 8 | Rocky surface | 10 | MOTORIZED |
| 9 | Bare soil | 20 | MOTORIZED |
| 10 | Mosaic of crops and fallows | 20 | MOTORIZED |
| 11 | Mosaic of crops and fallows under palm trees | 0 | MOTORIZED |
| 12 | Agglomeration | 30 | MOTORIZED |
| 13 | Water body (waterways) | 5 | WALKING |
| 14 | Sandy beach | 10 | MOTORIZED |
| 1001 | Primary roads | 80 | MOTORIZED |
| 1002 | Secondary roads | 30 | MOTORIZED |
| 1003 | Tracks | 20 | MOTORIZED |
| 1004 | Trails | 10 | MOTORIZED |

1. Scenario for Atacora and Donga departments

| **class** | **label** | **Speed (km/h)** | **mode** |
| --- | --- | --- | --- |
| 1 | Dense forest | 10 | MOTORIZED |
| 2 | Forest gallery | 10 | MOTORIZED |
| 3 | Clear forest and wooded savannah | 20 | MOTORIZED |
| 4 | Tree and shrub savannah | 20 | MOTORIZED |
| 5 | Bare and sparse vegetation | 10 | MOTORIZED |
| 6 | Swamp | 0 | MOTORIZED |
| 7 | Planting | 12 | MOTORIZED |
| 8 | Rocky surface | 5 | MOTORIZED |
| 9 | Bare soil | 20 | MOTORIZED |
| 10 | Mosaic of crops and fallows | 15 | MOTORIZED |
| 11 | Mosaic of crops and fallows under palm trees | 0 | WALKING |
| 12 | Agglomeration | 30 | MOTORIZED |
| 13 | Water body (waterways) | 0 | WALKING |
| 14 | Sandy beach | 0 | WALKING |
| 1001 | Primary roads | 80 | MOTORIZED |
| 1002 | Secondary roads | 40 | MOTORIZED |
| 1003 | Tracks | 30 | MOTORIZED |
| 1004 | Trails | 20 | MOTORIZED |

1. Scenario for Atlantique and Littoral departments

| **class** | **label** | **Speed (km/h)** | **mode** |
| --- | --- | --- | --- |
| 1 | Dense forest | 0 | WALKING |
| 2 | Forest gallery | 0 | WALKING |
| 3 | Clear forest and wooded savannah | 0 | WALKING |
| 4 | Tree and shrub savannah | 20 | MOTORIZED |
| 5 | Bare and sparse vegetation | 0 | WALKING |
| 6 | Swamp | 1 | WALKING |
| 7 | Planting | 10 | MOTORIZED |
| 8 | Rocky surface | 0 | WALKING |
| 9 | Bare soil | 0 | WALKING |
| 10 | Mosaic of crops and fallows | 20 | MOTORIZED |
| 11 | Mosaic of crops and fallows under palm trees | 20 | MOTORIZED |
| 12 | Agglomeration | 50 | MOTORIZED |
| 13 | Water body (waterways) | 10 | MOTORIZED |
| 14 | Sandy beach | 10 | MOTORIZED |
| 1001 | Primary roads | 80 | MOTORIZED |
| 1002 | Secondary roads | 40 | MOTORIZED |
| 1003 | Tracks | 30 | MOTORIZED |
| 1004 | Trails | 15 | MOTORIZED |

1. Scenario for Mono and Couffo departments

| **class** | **label** | **Speed (km/h)** | **mode** |
| --- | --- | --- | --- |
| 1 | Dense forest | 0 | WALKING |
| 2 | Forest gallery | 10 | MOTORIZED |
| 3 | Clear forest and wooded savannah | 20 | MOTORIZED |
| 4 | Tree and shrub savannah | 20 | MOTORIZED |
| 5 | Bare and sparse vegetation | 0 | WALKING |
| 6 | Swamp | 1 | WALKING |
| 7 | Planting | 10 | MOTORIZED |
| 8 | Rocky surface | 0 | WALKING |
| 9 | Bare soil | 0 | WALKING |
| 10 | Mosaic of crops and fallows | 20 | MOTORIZED |
| 11 | Mosaic of crops and fallows under palm trees | 20 | MOTORIZED |
| 12 | Agglomeration | 40 | MOTORIZED |
| 13 | Water body (waterways) | 10 | MOTORIZED |
| 14 | Sandy beach | 10 | MOTORIZED |
| 1001 | Primary roads | 80 | MOTORIZED |
| 1002 | Secondary roads | 40 | MOTORIZED |
| 1003 | Tracks | 30 | MOTORIZED |
| 1004 | Trails | 15 | MOTORIZED |

1. Scenario for Oueme and Plateau departments

| **class** | **label** | **Speed (km/h)** | **mode** |
| --- | --- | --- | --- |
| 1 | Dense forest | 0 | WALKING |
| 2 | Forest gallery | 10 | MOTORIZED |
| 3 | Clear forest and wooded savannah | 20 | MOTORIZED |
| 4 | Tree and shrub savannah | 20 | MOTORIZED |
| 5 | Bare and sparse vegetation | 0 | MOTORIZED |
| 6 | Swamp | 1 | WALKING |
| 7 | Planting | 10 | MOTORIZED |
| 8 | Rocky surface | 0 | WALKING |
| 9 | Bare soil | 0 | WALKING |
| 10 | Mosaic of crops and fallows | 20 | MOTORIZED |
| 11 | Mosaic of crops and fallows under palm trees | 20 | MOTORIZED |
| 12 | Agglomeration | 40 | MOTORIZED |
| 13 | Water body (waterways) | 10 | MOTORIZED |
| 14 | Sandy beach | 10 | MOTORIZED |
| 1001 | Primary roads | 80 | MOTORIZED |
| 1002 | Secondary roads | 40 | MOTORIZED |
| 1003 | Tracks | 30 | MOTORIZED |
| 1004 | Trails | 15 | MOTORIZED |

1. Scenario for Zou and Collines departments

| **class** | **label** | **Speed (km/h)** | **mode** |
| --- | --- | --- | --- |
| 1 | Dense forest | 0 | WALKING |
| 2 | Forest gallery | 10 | MOTORIZED |
| 3 | Clear forest and wooded savannah | 20 | MOTORIZED |
| 4 | Tree and shrub savannah | 20 | MOTORIZED |
| 5 | Bare and sparse vegetation | 0 | WALKING |
| 6 | Swamp | 0 | WALKING |
| 7 | Planting | 10 | MOTORIZED |
| 8 | Rocky surface | 0 | WALKING |
| 9 | Bare soil | 20 | MOTORIZED |
| 10 | Mosaic of crops and fallows | 20 | MOTORIZED |
| 11 | Mosaic of crops and fallows under palm trees | 0 | WALKING |
| 12 | Agglomeration | 40 | MOTORIZED |
| 13 | Water body (waterways) | 0 | MOTORIZED |
| 14 | Sandy beach | 0 | WALKING |
| 1001 | Primary roads | 80 | MOTORIZED |
| 1002 | Secondary roads | 40 | MOTORIZED |
| 1003 | Tracks | 30 | MOTORIZED |
| 1004 | Trails | 15 | MOTORIZED |
