# Supplementary material for: Optimization of the emergency obstetric and neonatal care network in Benin through expert-based sub-national prioritizations
Source: Front Glob Womens Health. 2024 Jun 3;5:1265729. doi: 10.3389/fgwh.2024.1265729 (PMC11180813; doi:10.3389/fgwh.2024.1265729)
Supplement: Supplementary file 2 [file Datasheet2.docx]

**Supplementary Figures and Tables**

Tables

**Supplementary Table 1** gives key information on all input data sets used in the accessibility analyses.

| **Themes** | **Year** | **Source name** | **URL for data download** |
| --- | --- | --- | --- |
| Population | 2014, adjusted to 2018 based on World Bank data growth rates | CIESIN/Data for Good at Meta | <https://data.humdata.org/dataset/highresolutionpopulationdensitymaps-ben> |
| Rivers | 2018 | Institut Géographique National du Bénin (IGN-BENIN) | <https://ign.bj/lign/> |
| Lakes | 2018 | Institut Géographique National du Bénin (IGN-BENIN) | <https://ign.bj/lign/> |
| Wet zones | 2018 | Institut Géographique National du Bénin (IGN-BENIN) | <https://ign.bj/lign/> |
| Roads | 2018 | Institut Géographique National du Bénin (IGN-BENIN) | <https://ign.bj/lign/> |
| Land cover | 2018 | Institut Géographique National du Bénin (IGN-BENIN) | <https://ign.bj/lign/> |
| DEM | 2018 | Institut Géographique National du Bénin (IGN-BENIN)  NASA's Shuttle Radar Topography Mission | <https://ign.bj/lign/> |
| Administrative boundaries | 2018 | Institut Géographique National du Bénin (IGN-BENIN) | <https://ign.bj/lign/> |
| Location of maternities | 2018 | UNFPA EmONC rapid assessment | Not publicly available |
